# Supplementary material for: ADCY5 Gene Affects Seasonal Reproduction in Dairy Goats by Regulating Ovarian Granulosa Cells Steroid Hormone Synthesis
Source: Int J Mol Sci. 2025 Feb 14;26(4):1622. doi: 10.3390/ijms26041622 (PMC11855216; doi:10.3390/ijms26041622)

Supplement Table 1

Supplement Table 1 Number of DEGs and parts of DEGs related to reproduction

Grouping	Number of up-regulated DEGs	Number of down-regulated DEGs	Parts of DEGs related to reproduction
LM1 vs M2	3542	2944	*STAR, 3BHSD, CYP11A1, EGFR, ADCY5, AKT3, LDLR, PIK3R1*and *MAPK12,* and so on.
C1 vs C2	930	608	*STAR, LDLR, PIK3R1，ADCY5, BMP15, FSHR,* and *CYP17A1* and so on.

Supplement Figure 1

Supplement Figure 1 The CDs sequence and the encoded protein sequence of *ADCY5*

1 ATGTCCAGCTCCAAAAGCGTGAGCCCCCCGGGCTACGCGGCGCAGACAGCGGCGGCGCCGGCGTCCCGGGGAGGCCCGGAACACCGCTCC

M S S S K S V S P P G Y A A Q T A A A P A S R G G P E H R S

91 GCGTGGGGAGAGGCCGACTCCCGCGCCAATGGCTACCCCCAAGCCCCCGGGGGTTCGGCCCGCGGCTCCACCAAGAGACCTGGGGGGGCG

A W G E A D S R A N G Y P Q A P G G S A R G S T K R P G G A

181 GTGACCCCGCAGCAGCAACAGCGCCTGGCCAGCCGCTGGCGCAGCGACGACGAAGACGATCCTCCGCTGAGTGGCGATGACCCCCTGGCC

V T P Q Q Q Q R L A S R W R S D D E D D P P L S G D D P L A

271 GGGGGCTTCGGCTTCAGCTTCCGTTCCAAGTCGGCCTGGCAGGAGCGCGGCGGCGACGACTGCGGTCGCGGCAGCCGGCGGCAGCGGCGG

G G F G F S F R S K S A W Q E R G G D D C G R G S R R Q R R

361 GGCGCGGCCGGCGGGGGCAGCACCCGGGCGCCCCCTGCGGGCAGCGGCGGCGGTTCGGCGGCTGCGGCCGCCGCAGCGGGAGGAACGGAG

G A A G G G S T R A P P A G S G G G S A A A A A A A G G T E

451 GTGCGCCCCCGTTCGGTGGAGCTAGGCCTGGAGGAGCGGCGTGGCAAGGGCCGCGCGGCCGAAGATCTGGAGGCCGGCGCCGTCGAGGAA

V R P R S V E L G L E E R R G K G R A A E D L E A G A V E E

541 GACGAAGGGTCCGGGGATGGGGGCAGCTCGGCAGGCTCGGGCTCGGGGCCTGGCGCGGTGCTGTCGCTGGGCGCCTGCTGCCTGGCACTG

D E G S G D G G S S A G S G S G P G A V L S L G A C C L A L

631 CTGCAGATATTCCGCTCGAAGAAGTTCCCATCGGACAAGCTGGAGCGGCTGTACCAGCGCTACTTCTTCCGTCTGAACCAGAGCAGCCTC

L Q I F R S K K F P S D K L E R L Y Q R Y F F R L N Q S S L

721 ACGATGCTCATGGCTGTGCTGGTGCTCGTGTGCCTAGTCATGTTGGCCTTCCACGCCGCACGGCCCCCGCTGCAGCTGCCCTATCTGGCC

T M L M A V L V L V C L V M L A F H A A R P P L Q L P Y L A

811 GTCCTAGCGGCCGCCGTGGGTGTGATCCTCGTTATGGCCGTGCTCTGCAACCGTGCCGCCTTCCACCAGGACCACATGGGCCTGGCCTGC

V L A A A V G V I L V M A V L C N R A A F H Q D H M G L A C

901 TACGCGCTTATCGCCGTGGTGCTGGCCGTCCAGGTGGTGGGCCTGCTGCTGCCCCAGCCGCGCAGTGCCTCCGAGGGCATCTGGTGGACT

Y A L I A V V L A V Q V V G L L L P Q P R S A S E G I W W T

991 GTGTTTTTCATCTATACGATCTACACGCTCCTGCCCGTGCGCATGCGGGCTGCGGTACTCAGCGGGGTGCTCCTGTCCGCCCTCCACCTG

V F F I Y T I Y T L L P V R M R A A V L S G V L L S A L H L

1081 GCCATCGCCCTGCGCACCAACGCCCAGGATCAGTTCCTGCTCAAGCAGCTCGTCTCCAATGTCCTCATTTTCTCCTGCACCAACATCGTG

A I A L R T N A Q D Q F L L K Q L V S N V L I F S C T N I V

1171 GGTGTGTGCACCCACTACCCGGCTGAGGTCTCGCAGAGACAGGCCTTCCAGGAGACCCGGGAGTGCATCCAGGCGCGGCTCCACTCGCAG

G V C T H Y P A E V S Q R Q A F Q E T R E C I Q A R L H S Q

1261 CGGGAGAACCAGCAGCAGGAGCGGCTCCTGCTGTCTGTCCTTCCCCGTCACGTTGCCATGGAGATGAAAGCTGACATCAACGCCAAACAG

R E N Q Q Q E R L L L S V L P R H V A M E M K A D I N A K Q

1351 GAGGATATGATGTTCCATAAGATTTACATCCAGAAACATGACAACGTGAGCATCCTGTTTGCTGACATCGAGGGTTTCACCAGCCTGGCA

E D M M F H K I Y I Q K H D N V S I L F A D I E G F T S L A

1441 TCCCAGTGCACTGCCCAGGAGCTGGTCATGACCCTCAATGAGCTCTTCGCCCGCTTCGACAAGCTGGCTGCGGAGAATCACTGTTTACGT

S Q C T A Q E L V M T L N E L F A R F D K L A A E N H C L R

1531 ATTAAGATCCTGGGAGATTGTTATTACTGTGTCTCAGGGCTGCCTGAAGCCAGGGCTGACCACGCCCACTGCTGCGTGGAGATGGGCATG

I K I L G D C Y Y C V S G L P E A R A D H A H C C V E M G M

1621 GACATGATCGAGGCCATCTCGCTGGTCCGGGAGGTGACAGGTGTGAACGTGAACATGCGTGTGGGAATTCACAGCGGGAGAGTGCACTGC

D M I E A I S L V R E V T G V N V N M R V G I H S G R V H C

1711 GGTGTCCTCGGCCTCAGGAAGTGGCAGTTCGATGTCTGGTCTAACGATGTCACACTGGCCAACCACATGGAGGCTGGAGGCAAGGCGGGA

G V L G L R K W Q F D V W S N D V T L A N H M E A G G K A G

1801 CGCATCCACATCACCAAGGCCACACTCAACTACTTGAATGGTGACTACGAGGTGGAGCCAGGCTGTGGGGGCGAGCGCAACGCCTACCTC

R I H I T K A T L N Y L N G D Y E V E P G C G G E R N A Y L

1891 AAGGAGCACAGTATCGAGACCTTTCTCATCCTGCGCTGCACCCAGAAGCGGAAAGAAGAAAAGGCCATGATTGCCAAGATGAACCGCCAG

K E H S I E T F L I L R C T Q K R K E E K A M I A K M N R Q

1981 AGAACCAACTCCATCGGGCACAACCCGCCTCACTGGGGGGCCGAGCGCCCCTTCTATAACCACCTGGGTGGCAACCAGGTGTCCAAGGAG

R T N S I G H N P P H W G A E R P F Y N H L G G N Q V S K E

2071 ATGAAGCGGATGGGCTTTGAAGACCCCAAGGACAAGAATGCCCAGGAAAGTTCGAACCCTGAGGATGAAGTGGATGAATTTCTGGGCCGT

M K R M G F E D P K D K N A Q E S S N P E D E V D E F L G R

2161 GCCATTGATGCCAGGAGCATCGACAGGCTGCGATCCGAACATGTGCGCAAGTTCCTCTTGACCTTCAGGGAGCCTGACTTAGAGAAGAAG

A I D A R S I D R L R S E H V R K F L L T F R E P D L E K K

2251 TACTCCAAGCAAGTGGATGACCGATTCGGTGCCTACGTGGCCTGTGCCTCGCTCGTCTTCCTCTTCATCTGCTTTGTCCAGATCACCGTC

Y S K Q V D D R F G A Y V A C A S L V F L F I C F V Q I T V

2341 GTGCCCCACTCCGTGTTCATGTTGAGTTTCTACCTGACCTGTTTCCTGCTGCTGACCTTGGTGGTGTTTGTGTCCGTGATCTATTCTTGC

V P H S V F M L S F Y L T C F L L L T L V V F V S V I Y S C

2431 GTGAAGCTCTTCCCGGCCCCGCTGCAGACCCTCTCCAGGAAGATCGTGAGGTCCAAGATGAACAGCACCCTAGTGGGGGTGTTCACCATC

V K L F P A P L Q T L S R K I V R S K M N S T L V G V F T I

2521 ACCCTCGTGTTCCTGTCGGCTTTCGTCAACATGTTCACGTGCAACTCCAAGGACCTGTTTGGCTGCCTGGGGGACGAGCACAACATCAGT

T L V F L S A F V N M F T C N S K D L F G C L G D E H N I S

2611 GCCAGTCAGGTCAACGCGTGCCACGTGGTGGAGTCGGCCGTCAACTACAGTCTGGGTGATGAGCAGGGCTTCTGCGGCAGCTCCTGGCCC

A S Q V N A C H V V E S A V N Y S L G D E Q G F C G S S W P

2701 AACTGCAACTTCCCCGAGTACTTCACCTACAGCGTGCTGCTCAGCCTGCTGGCCTGCTCCGTGTTCCTGCAGATCAGCTGCATCGGGAAG

N C N F P E Y F T Y S V L L S L L A C S V F L Q I S C I G K

2791 CTGGTGCTCATGCTGGCCATCGAGCTCATCTACGTGCTCGTCGTTGAGGTGCCCGGTGTCACACTCTTTGACAATGCCGACCTCCTGGTC

L V L M L A I E L I Y V L V V E V P G V T L F D N A D L L V

2881 ACGGCCAACGCCATAGACTTCAACAACAACGGGACCTCCCAGTGCCCTGAGCATCCGACCAAGGTGGCGCTGAAGGTGGTGACGCCCGTC

T A N A I D F N N N G T S Q C P E H P T K V A L K V V T P V

2971 ATCATCTCTGTCTTCGTGCTGGCCCTGTACCTGCACGCCCAGCAGGTGGAGTCCACTGCCCGCCTCGACTTCCTCTGGAAACTTCAGGCC

I I S V F V L A L Y L H A Q Q V E S T A R L D F L W K L Q A

3061 ACAGAGGAGAAGGAGGAGATGGAGGAGCTGCAGGCTTACAACCGGCGGCTGCTGCACAATATTCTGCCCAAGGACGTGGCCGCACACTTC

T E E K E E M E E L Q A Y N R R L L H N I L P K D V A A H F

3151 CTGGCCCGTGAACGGCGCAACGACGAGCTCTACTATCAGTCGTGCGAGTGCGTGGCCGTCATGTTCGCCTCCATCGCCAACTTCTCCGAG

L A R E R R N D E L Y Y Q S C E C V A V M F A S I A N F S E

3241 TTCTACGTGGAACTGGAGGCCAACAACGAGGGCGTCGAGTGCCTGCGGCTGCTCAACGAGATCATCGCAGACTTTGACGAGATCATTAGT

F Y V E L E A N N E G V E C L R L L N E I I A D F D E I I S

3331 GAGGACCGGTTCAGGCAGCTGGAAAAGATCAAGACCATCGGCAGCACCTACATGGCCGCCTCGGGCCTCAATGACTCCACCTACGACAAG

E D R F R Q L E K I K T I G S T Y M A A S G L N D S T Y D K

3421 GTGGGCAAGACCCACATCAAAGCCCTGGCCGACTTCGCCATGAAGTTGATGGACCAAATGAAGTACATCAACGAGCACTCTTTCAACAAC

V G K T H I K A L A D F A M K L M D Q M K Y I N E H S F N N

3511 TTCCAGATGAAGATCGGGCTCAATATTGGCCCCGTGGTGGCGGGCGTGATCGGAGCCCGCAAGCCTCAGTACGACATCTGGGGCAATACG

F Q M K I G L N I G P V V A G V I G A R K P Q Y D I W G N T

3601 GTGAACGTGGCCAGCCGCATGGACAGCACCGGTGTGCCCGACCGCATCCAGGTCACCACAGACATGTACCAGGTGCTGGCCGCCAACACG

V N V A S R M D S T G V P D R I Q V T T D M Y Q V L A A N T

3691 TACCAGCTGGAGTGCCGGGGTGTGGTCAAGGTCAAGGGCAAAGGCGAGATGATGACCTACTTCCTCAACGGAGGGCCCCCGCCCAGTTAG

Y Q L E C R G V V K V K G K G E M M T Y F L N G G P P P S *

Supplement Figure 2. The effects of Br-cAMP on the proliferation of GCs and the phosphorylation levels of p38 and CREB.

(A)Effects of Br-cAMP addition on the proliferation of GCs. (B) Phosphorylation levels of CREB and p38 proteins in GCs after Br-cAMP addition. Data are shown as mean ± SEM for 3 independent replicates. * represents $P<0.05$; ** represents $P<0.01$; *** represents $P<0.001$.

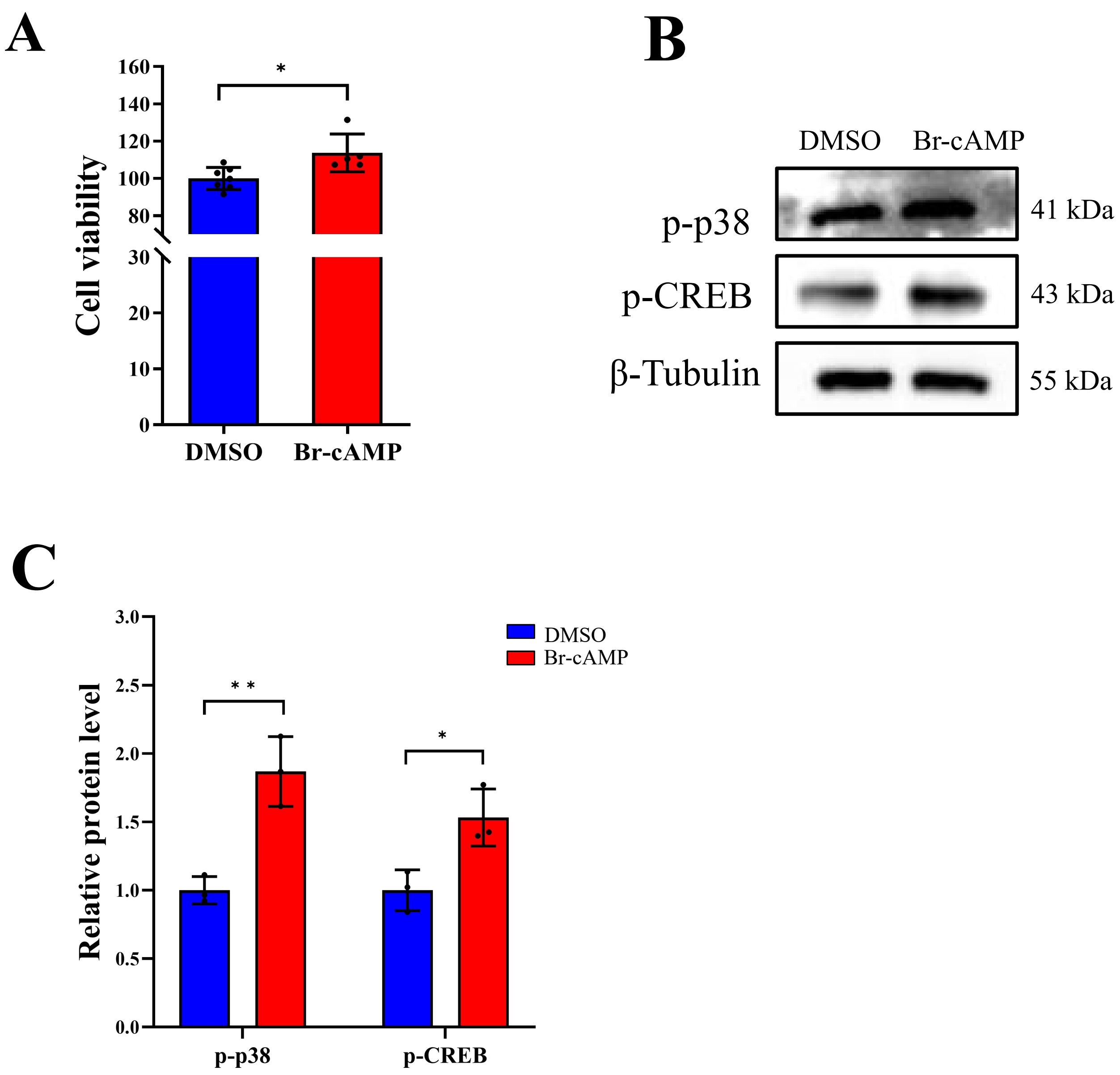

Supplement: Supplementary file 1 [file ijms-26-01622-s001.zip › ijms-3421305-supplementary.pdf]
